# Historical translocations and stocking alter the genetic structure of a Mediterranean lobster fishery

**DOI:** 10.1002/ece3.6304

**Published:** 2020-04-28

**Authors:** Tom L. Jenkins, Charlie D. Ellis, Eric D. H. Durieux, Jean‐José Filippi, Jérémy Bracconi, Jamie R. Stevens

**Affiliations:** ^1^ Molecular Ecology and Evolution Group Department of Biosciences College of Life and Environmental Sciences University of Exeter Exeter UK; ^2^ UMR CNRS 6134 Sciences Pour l’Environnement Università di Corsica Pasquale Paoli Corte France; ^3^ UMS CNRS 3514 STELLA MARE Università di Corsica Pasquale Paoli Biguglia France

**Keywords:** assignment, fisheries, genetic structure, hatchery, lobster, Mediterranean, single nucleotide polymorphisms, SNP panel, stocking, translocation

## Abstract

Stocking is often used to supplement wild populations that are overexploited or have collapsed, yet it is unclear how this affects the genetic diversity of marine invertebrate populations. During the 1970s, a lobster stock enhancement program was carried out around the island of Corsica in the Mediterranean using individuals translocated from the Atlantic coast of France. This included the release of thousands of hatchery‐reared postlarval lobsters and several adult individuals, but no monitoring plan was established to assess whether these animals survived and recruited to the population. In this study, we sampled European lobster (*Homarus gammarus*) individuals caught around Corsica and tested whether they showed Atlantic ancestry. Due to a natural marked phylogeographic break between Atlantic and Mediterranean lobsters, we hypothesized that lobsters with dominant (>0.50) Atlantic ancestry were descended from historical stocking releases. Twenty Corsican lobsters were genotyped at 79 single nucleotide polymorphisms, and assignment analysis showed that the majority (13) had dominant Atlantic ancestry. This suggests that the hatchery stocking program carried out in Corsica during the 1970s, using individuals translocated from the Atlantic coast of France, has likely augmented local recruitment but at a cost of altering the genetic structure of the Corsican lobster population.

## Graphical abstract

Stocking is often used to supplement wild populations that are overexploited or have collapsed, yet it is unclear how this affects the genetic diversity of marine invertebrate populations. In this study, European lobsters caught around Corsica in the Mediterranean were genotyped and the results showed that a large proportion of lobsters have dominant Atlantic ancestry. This suggests that the stock enhancement programme carried out in Corsica during the 1970s, using individuals translocated from the Atlantic coast of France, has likely augmented recruitment but at a cost of altering the genetic structure of the Corsican lobster population.

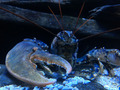

## 1 INTRODUCTION

**FIGURE 1:**
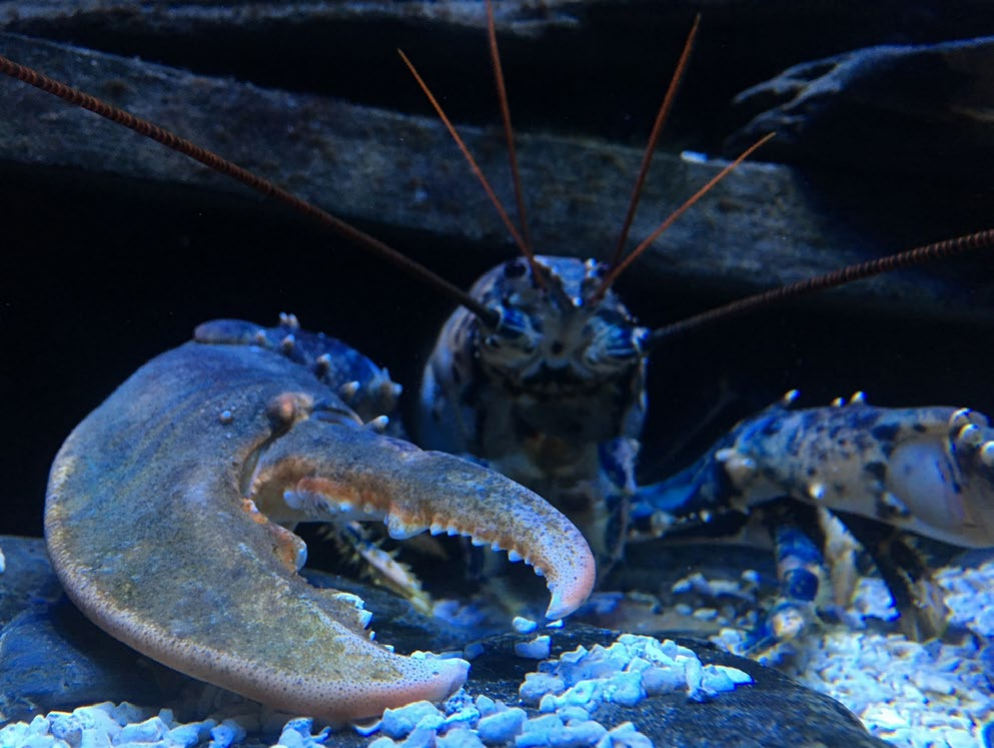
An adult male European lobster (*Homarus gammarus*; Credit: Chris Weston)

Global fisheries are under intense pressure, with many stocks overexploited or near collapse (Pauly & Zeller, 2016;Worm et al., 2009). In the case of the economically valuable European lobster (*Homarus gammarus*; Figure 1), overfishing during the middle of the twentieth century caused stock collapses throughout Scandinavia (Agnalt, Kristiansen, & Jørstad, 2007;Dow, 1980), where stocks generally remain at <10% of precollapse levels (Kleiven, Olsen, & Vølstad, 2012), and historical overexploitation similarly decimated Mediterranean populations (Spanier et al., 2015). Such stock declines at both regional and local scales have led to the rearing of *H. gammarus* larvae in lobster hatcheries to produce juveniles which are released to supplement wild stocks (Bannister & Addison, 1998;Ellis et al., 2015). However, there is still uncertainty as to whether lobster stock enhancement programs are worthwhile.

Part of this uncertainty arises from the challenge of identifying hatchery‐reared lobsters once they are admixed with natural stocks in the wild. A few approaches have been employed to distinguish hatchery lobsters from their wild counterparts; typically, these involve the insertion of small internal tags that are retained through ecdysis (Ellis et al., 2015). However, these approaches have relied on using ~1‐year‐old animals because tag retention is poor in younger juveniles (Ellis et al., 2015). This has provided proof of principle for stocking via identifiable recaptures, but it is more costly to rear juveniles to this age, which has resulted in a failure to demonstrate evidence of economic viability (Ellis et al., 2015). To maximize release numbers and to minimize costs, recent lobster stocking ventures have instead tended to release juveniles as soon as they are fully benthic (stage V–VI; 1–2 months old), despite this largely invalidating traditional physical tagging options to monitor their fate in the wild (Ellis et al., 2015). Therefore, any data which allow fisheries managers and scientists to infer long‐term survival of released postlarval juveniles represent a valuable contribution toward assessing the viability of current lobster stock enhancement programs.

**FIGURE 2:**
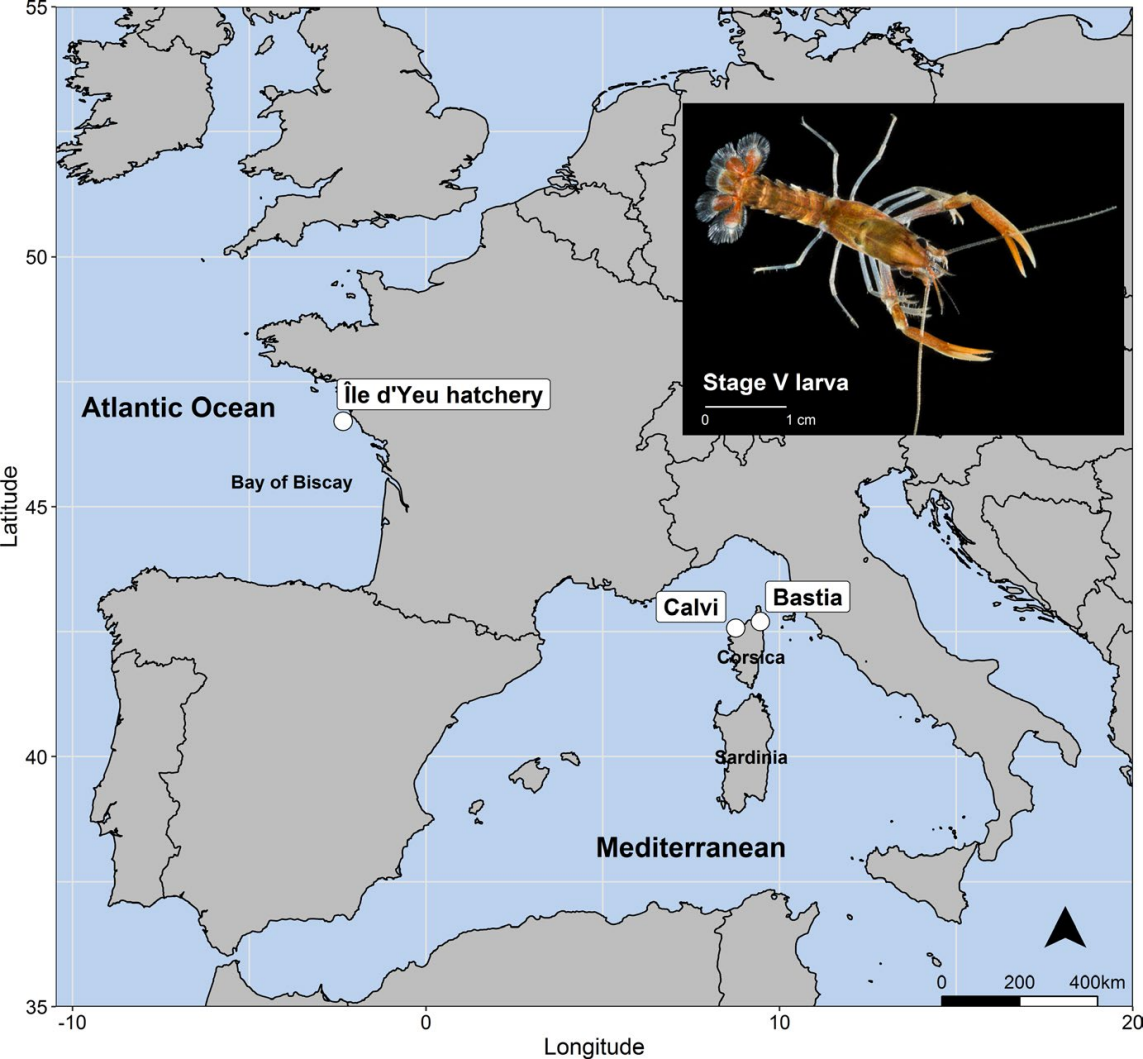
Map showing the location of the Île d'Yeu lobster hatchery in the Bay of Biscay and the location of the two sites in Corsica that were stocked during the 1970s: Bastia and Calvi. The top‐right image depicts a hatchery‐reared European lobster postlarval juvenile at instar stage V, at least ten thousand of which were released in Corsica over 40 years ago (Credit: Alex Hyde)

Such a program was conducted in France during the 1970s with over 1 million lobster juveniles produced and released along the French coasts (Audouin, 1979). Juveniles were produced from a hatchery located on the Île d'Yeu in the Bay of Biscay region of the northeast Atlantic (Figure 2). While the program released juveniles mainly in Atlantic sites, releases were also conducted in Corsica, a French island located in the central Mediterranean (Figure 2), using the same hatchery‐reared juvenile lobsters originating from egg‐bearing females sourced locally around Île d'Yeu (Audouin, 1979). In total, 10,200 stage V–VI juveniles of Atlantic origin were released around Bastia (northeast Corsica) in 1976 (Audouin, 1979), with a further 600 juveniles and several adults purportedly originating from Île d'Yeu released around Calvi (northwest Corsica) in 1978 (Appendix S1).

Highly accurate genetic assignment of European lobsters to their ocean basin of origin (either the Atlantic or the Mediterranean) has recently been demonstrated (Jenkins, Ellis, Triantafyllidis, & Stevens, 2019) using a genetic marker panel comprised of 79 single nucleotide polymorphisms (SNPs; Jenkins, Ellis, & Stevens, 2019). The availability of this stock identification tool presents an opportunity to test whether there are any Atlantic genetic signatures remaining among contemporary Corsican lobsters. Therefore, we generated SNP genotypes for lobsters caught around the coast of Corsica from 2013 to 2020 and used the baseline data set and assignment analyses outlined in Jenkins, Ellis, Triantafyllidis, et al. (2019) to predict whether they are descended from Atlantic stocks. We hypothesized that because of the pronounced phylogeographic break found between Atlantic and Mediterranean lobsters (Jenkins, Ellis, Triantafyllidis, et al., 2019), lobsters caught locally in Corsica that assigned predominantly (>0.50) as Atlantic were derived or descended from the stocking carried out during the 1970s.

## 2 MATERIALS AND METHODS

### 2.1 Sampling, SNP genotyping, and quality control

Telson samples from adult European lobsters were collected during 2013–2020 around Corsica from local artisanal fishers and preserved in 95%–100% ethanol. DNA was extracted using a salting‐out protocol (Jenkins, Ellis, & Stevens, 2019), and the concentration and quality of DNA were quantified using a NanoDrop One spectrophotometer. SNP genotyping was carried out using a Fluidigm EP1 System as per Jenkins, Ellis, Triantafyllidis, et al. (2019), and genotypes were called using the Fluidigm SNP Genotyping Analysis software. Quality control and filtering also followed the methods of Jenkins, Ellis, Triantafyllidis, et al. (2019). Briefly, a missing data threshold of 30% was enforced and 17 loci from the original panel of 96 SNPs were removed from the data set due to poor genotyping consistency, abnormal heterozygosity patterns, or departures from Hardy–Weinberg equilibrium or linkage equilibrium.

### 2.2 Data analysis

To visualize the genetic clustering of Corsican lobsters, together with the full data set from Jenkins, Ellis, Triantafyllidis, et al. (2019) which comprised 1,278 individuals from 38 sampling sites across the species’ range (available from Dryad; https://doi.org/10.5061/dryad.2v1kr38), a discriminant analysis of principal components (DAPC) was performed using the *dapc* function from adegenet v2.1.1 (Jombart & Ahmed, 2011) implemented in R (R Core Team, 2020). Cross‐validation using the *xvalDapc* function from adegenet was used to choose the optimal number of principal components to retain.

To predict the origin of the lobsters caught in Corsica, the basin of origin baseline data set from Jenkins, Ellis, Triantafyllidis, et al. (2019) was downloaded from Dryad. Next, the SNP genotypes of the Corsican lobsters were exported to a separate file in genepop format. Then, both data sets were used as input files to the *assign.X* function from assignPOP v1.1.4 (Chen et al., 2018), which uses the baseline data (i.e., the basin of origin data set) to build a predictive model which is subsequently used to make predictions on the origin of the test data (i.e., the genotypes of the Corsican lobsters). To ensure consistency between data sets, the support vector machine classification function was also used in the model in this study. To check for introgression of Atlantic‐associated alleles into adjacent areas, assignment to basin of origin was repeated for the nearest geographic sample, Sardinia, with 22 individuals sourced from 2013 to 2017 being removed from the baseline data set and then run as the test data against the revised baseline.

## 3 RESULTS

**FIGURE 3:**
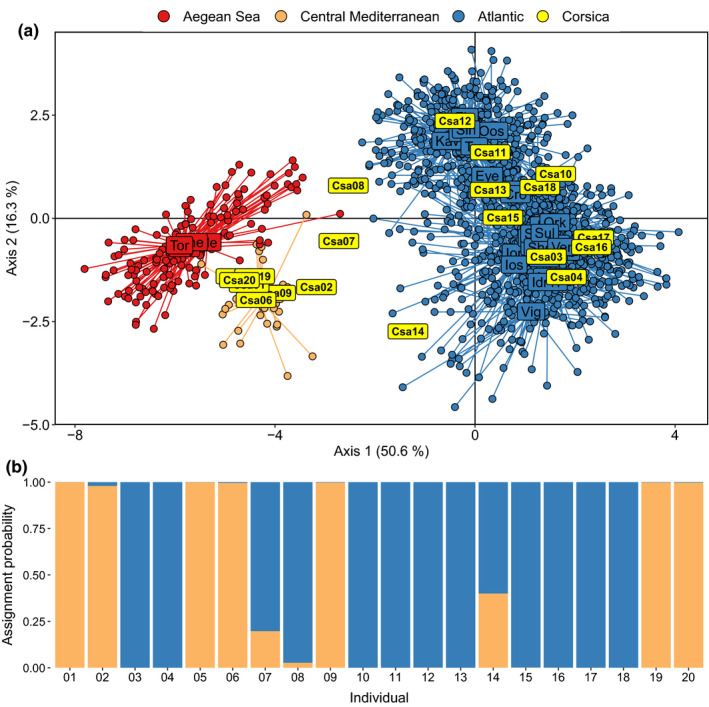
Analysis of Corsican lobster genotypes based on 79 single nucleotide polymorphisms. (a) Discriminant analysis of principal components. Circular points represent individuals, and colors denote the region of origin of each individual. The positions of the Corsican lobsters are represented by numbers within rectangular yellow labels. (b) Assignment probabilities of Corsican lobsters. Colors correspond to ocean basin of origin: Atlantic is denoted by blue, and (central) Mediterranean is denoted by orange

After quality control and filtering, all 20 lobsters were retained for downstream analyses. A DAPC showed that seven of these lobsters grouped with a central Mediterranean cluster composed of Sardinia and Lazio baseline samples, while three Corsican individuals were placed between the main Atlantic and Mediterranean clusters; the remaining 10 Corsican individuals grouped within the Atlantic cluster (Figure 3a). These 10 individuals were positioned variably across the Atlantic genetic cline, grouping with individuals sampled from around Atlantic France, Britain, Ireland, and Scandinavia.

In the assignment tests, the model predicted the seven Corsican individuals that grouped in the central Mediterranean cluster in the DAPC to be of Mediterranean descent (Figure 3b). The remaining Corsican individuals were predicted to be of majority Atlantic descent, although two of the individuals (Csa07 and Csa14) that were positioned between clusters in the DAPC also showed mixed membership between Atlantic and Mediterranean lineages via assignment. In contrast, all Sardinian individuals assigned strongly to the Mediterranean basin (Appendix S2).

## 4 DISCUSSION

The results of this study suggest that translocation and stocking during the 1970s led to the successful establishment of released lobsters, but have also altered the genetic structure of the Corsican lobster population. Among 20 lobsters from Corsica, seven individuals assigned predominantly to the Mediterranean and these likely descend solely from native populations. The remaining lobsters assigned predominantly to the Atlantic which suggests that these individuals are either the animals translocated in the 1970s or their descendants. Techniques to precisely age lobsters remain elusive, but studies of longevity suggest that European lobsters can have life spans in excess of the 44‐year maximum duration between Corsican stocking and the sampling for this study (Sheehy, Bannister, Wickins, & Shelton, 1999). Based on the broad range of body sizes recorded (Table 1), and assuming a general correlation between size and age (Bannister & Addison, 1998), it is not implausible that the largest Atlantic‐assigning individuals (>2 kg) in our sample could be direct survivors from the original postlarval stocking releases. Similarly, it is likely that the smaller of our samples (individuals < 1 kg) are at least one generation removed from the original stocking event. Given that the vast majority of lobsters released in Corsica were postlarval juveniles, the consistent presence of Atlantic‐assigning lobsters in the contemporary Corsican population presents the strongest evidence yet that hatchery‐reared European lobster postlarvae (stages V and VI) can survive to adulthood in the wild.

**TABLE 1:** Information for each lobster locally sampled in Corsica

ID	Sample date	Location	Depth (m)	Sex	Carapace length (mm)	Weight (kg)	Assignment probability	
							Atlantic	Mediterranean
Csa01	01‐07‐2013	Centuri		Male	120	1.30	0.0003	0.9997
Csa02	01‐07‐2013	Centuri		Male	130	1.60	0.0205	0.9795
Csa03	04‐07‐2013	Saint‐Florent		Male	96	0.60	1.0000	0.0000
Csa04	04‐07‐2013	Saint‐Florent		Male	95	0.60	0.9999	0.0001
Csa05	01‐07‐2014	Centuri		Male	205	3.38	0.0013	0.9987
Csa06	02‐07‐2014	Centuri		Male	155	1.36	0.0046	0.9954
Csa07	10‐09‐2015	Bastia		Female	180	2.30	0.8029	0.1971
Csa08	17‐12‐2015	Figari		Female	195	2.80	0.9730	0.0270
Csa09	21‐12‐2015	Porto‐Vecchio		Female	183	2.56	0.0020	0.9980
Csa10	05‐02‐2016	Bastia (Barge Marana Wreck)	54	Female	165	1.70	1.0000	0.0000
Csa11	06‐02‐2016	Bastia (FIAT RS14 Wreck)	72	Female	168	1.70	1.0000	0.0000
Csa12	09‐02‐2016	Pietracorbara (Insuma Wreck)	43	Female	174	1.80	0.9992	0.0008
Csa13	09‐02‐2016	Pietracorbara (Insuma Wreck)	43	Female	177	2.00	0.9999	0.0001
Csa14	27‐02‐2017	Bastia (FIAT RS14 Wreck)	72	Female	126	0.71	0.6000	0.4000
Csa15	27‐02‐2017	Bastia (FIAT RS14 Wreck)	72	Female	130	0.74	0.9985	0.0015
Csa16	28‐02‐2017	Bastia (FIAT RS14 Wreck)	72	Female	115	0.56	0.9997	0.0003
Csa17	28‐02‐2017	Bastia (FIAT RS14 Wreck)	72	Female	135	0.75	0.9999	0.0001
Csa18	2018	Bastia		Male	160	1.50	1.0000	0.0000
Csa19	17‐08‐2018	Calvi		Female	193	1.35	0.0019	0.9981
Csa20	12‐01‐2020	Calvi		Female	200	2.92	0.0029	0.9971

Significantly, the presence of a dominant Atlantic signature in 13/20 lobsters sampled implies that Atlantic‐origin lobsters translocated into Corsican waters established to the extent that they or their descendants now comprise a major component of the local fishery. Moreover, this Atlantic signature is highly unlikely to be an artefact of natural connectivity between the Atlantic and the Mediterranean because of the distinct phylogeographic break observed between lobsters of the two ocean basins (Jenkins, Ellis, Triantafyllidis, et al., 2019); this is further supported by a distinct lack of any major Atlantic genetic signature in lobsters from nearby Sardinia (Appendix S2). Thus, the impacts of stocking postlarval lobsters on local abundance, and the impacts of distantly relocating individuals beyond the extent of natural connectivity on population genetic structure, can both be extensive and long‐lasting. Such a finding has been reported in several fish species (Araki, Cooper, & Blouin, 2007;Gonzalez, Aritaki, Knutsen, & Taniguchi, 2015) and was linked to reduced fitness of the natural population in steelhead trout (Araki et al., 2007), but is largely unstudied in marine invertebrate populations.

Of the Corsican lobsters that had a distinct Atlantic signature, these individuals were positioned across the southern (Atlantic France, Ireland and Britain) and northern (Scandinavian) portion of the Atlantic genetic cline (Figure 3a). Given the high genetic connectivity reported in lobster populations across the northeast Atlantic (Jenkins, Ellis, Triantafyllidis, et al., 2019), the spread of Île d'Yeu‐origin lobsters across the northeast Atlantic cline is broadly as anticipated. In addition, the two Corsica‐caught lobsters that showed mixed assignment broadly fit the expected profiles of lobsters with introgressed Atlantic and Mediterranean ancestry. For instance, individual Csa14 fits F_1_ expectations of direct mating between one Atlantic and one Mediterranean parent, while Csa07 fits an F_2_ profile resulting from a mixed‐lineage F_1_ backcrossing with a wholly Atlantic parent (Figure 3b). Such introgression may have inadvertent ramifications for the genetic diversity, structure, and adaptive fitness of Corsican stocks. However, it is not clear how the stocking outlined in this study may have affected the demography and the population genetics of lobster stocks around Corsica, or how readily these effects may disperse to stocks in adjacent regions such as Sardinia, western Italy, and southern France in the longer term. Regardless, the genetic tool and the analysis pipeline used in this study provide an opportunity to assess future changes in stock structure in Corsica and enable the potential spillover of genetic variants to neighboring Mediterranean stocks to be monitored. Our results also reinforce the need for hatchery stocking operations to consider the compatibility of released stock with the genetic structure of any wild populations to be augmented.

Centuries of overexploitation have led to collapses in European lobster stocks across the Mediterranean (Spanier et al., 2015), with only artisanal fisheries remaining in many areas (Pere, Marengo, Lejeune, & Durieux, 2019). This study demonstrates the utility of a genetic marker panel to detect lobsters of Atlantic descent in Corsica and provides evidence that a number of translocated lobsters of Atlantic origin survived and have contributed to recruitment in the wild. Furthermore, the results suggest that restocking small‐scale fisheries such as Corsica with hatchery‐reared juveniles can be beneficial in terms of enhancing local abundance, but also that translocating animals beyond the extent of natural connectivity can have profound and long‐lasting impacts on the genetic makeup of admixed stocks. This system offers a unique opportunity to assess how stocking affects the evolutionary trajectory of a marine invertebrate population, which can be critical for managers but is currently not well known for many stocked fisheries.

## CONFLICT OF INTEREST

The authors declare no competing interests.

## AUTHOR CONTRIBUTION

**Tom Jenkins:** Conceptualization (equal); Formal analysis (equal); Visualization (lead); Writing‐original draft (lead); Writing‐review & editing (equal). **Charlie Ellis:** Conceptualization (equal); Formal analysis (equal); Writing‐review & editing (equal). **Eric Durieux:** Conceptualization (equal); Data curation (equal); Writing‐review & editing (equal). **Jean‐José Filippi:** Data curation (equal); Writing‐review & editing (supporting). **Jérémy Bracconi:** Data curation (equal); Writing‐review & editing (supporting). **Jamie Stevens:** Conceptualization (equal); Funding acquisition (lead); Writing‐review & editing (equal).

## Supporting information

Appendix S1

Appendix S2

## Data Availability

All novel genotypes and R scripts are archived in the Dryad Digital Repository (https://doi.org/10.5061/dryad.cvdncjt1d).
