# Supplementary figures and images for: Historical translocations and stocking alter the genetic structure of a Mediterranean lobster fishery
Source: Ecol Evol. 2020 Apr 28;10(12):5631–6. doi: 10.1002/ece3.6304 (PMC7319110; doi:10.1002/ece3.6304)

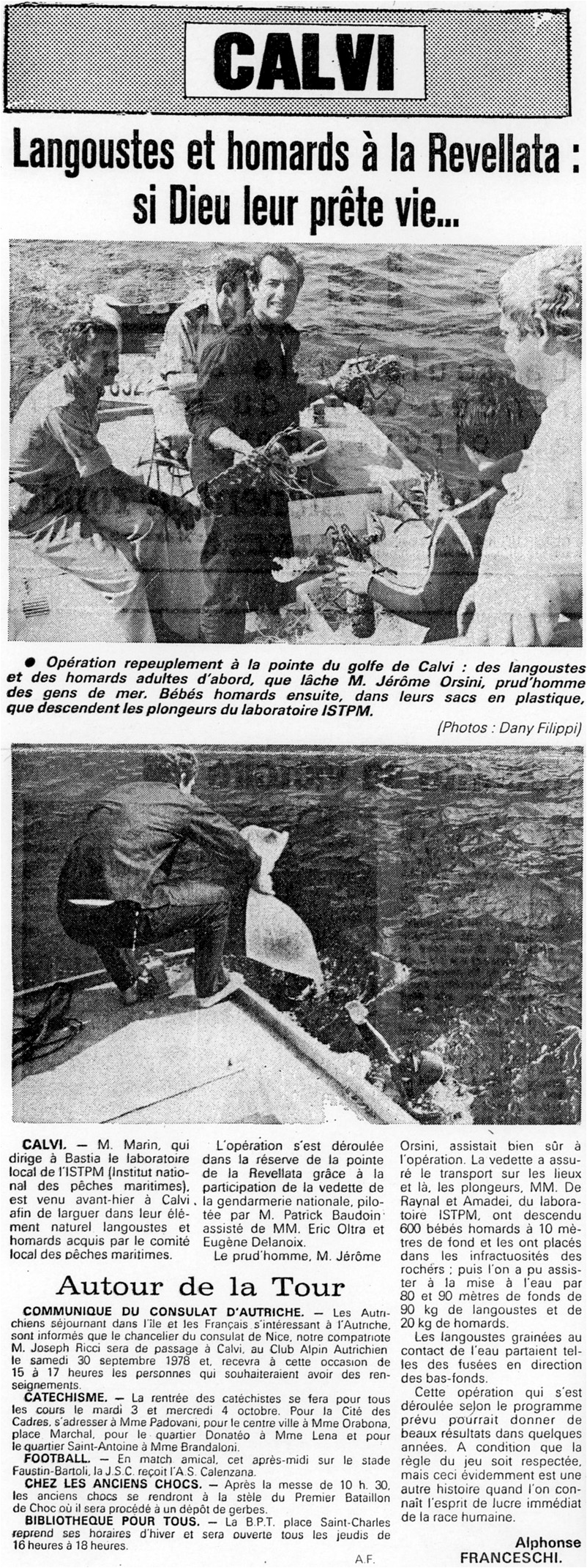

Supplement: Supplementary file 1 — Appendix S1 [file ECE3-10-5631-s001.png]

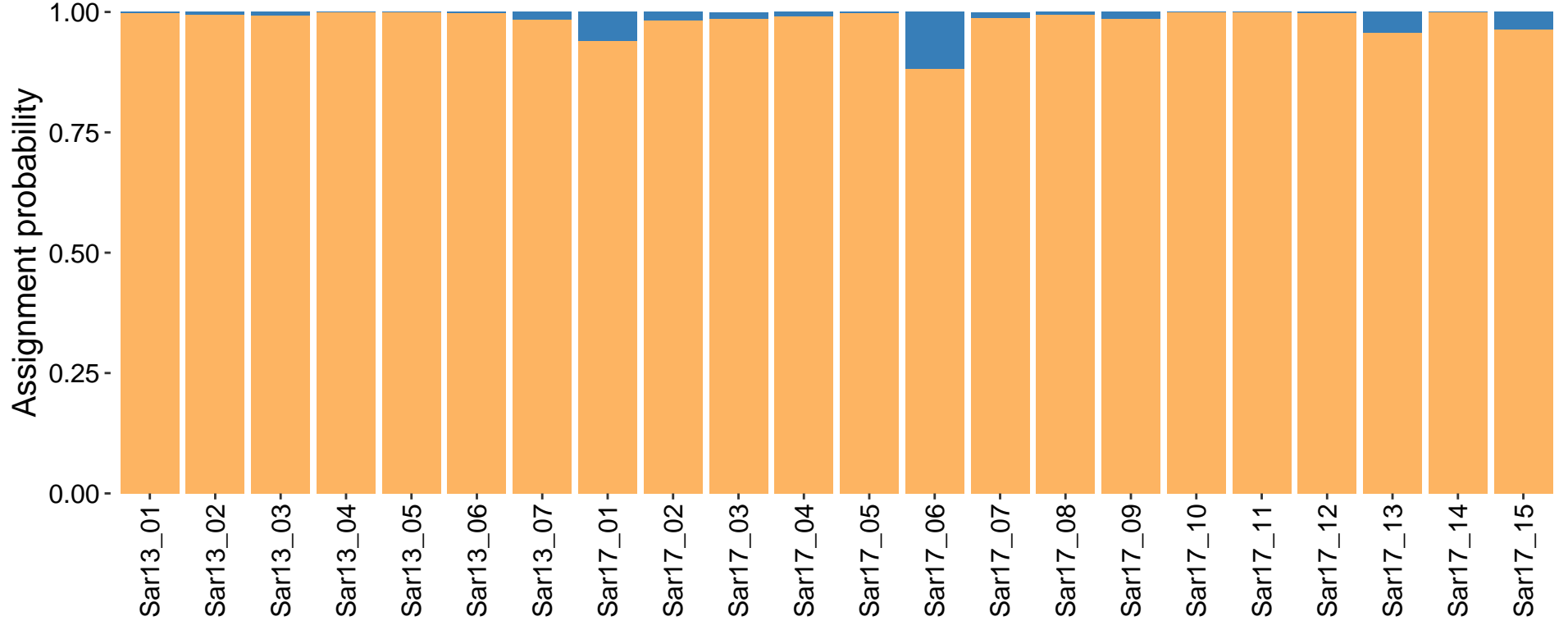

Supplement: Supplementary file 2 — Appendix S2 [file ECE3-10-5631-s002.pdf]
